# Pre-stimulus Alpha Activity Modulates Face and Object Processing in the Intra-Parietal Sulcus, a MEG Study

**DOI:** 10.3389/fnhum.2022.831781

**Published:** 2022-05-02

**Authors:** Narjes Soltani Dehaghani, Burkhard Maess, Reza Khosrowabadi, Reza Lashgari, Sven Braeutigam, Mojtaba Zarei

**Affiliations:** ^1^Institute of Medical Science and Technology, Shahid Beheshti University, Tehran, Iran; ^2^Institute for Cognitive and Brain Sciences, Shahid Beheshti University, Tehran, Iran; ^3^Max Planck Institute for Human Cognitive and Brain Sciences, Leipzig, Germany; ^4^Oxford Centre for Human Brain Activity, Wellcome Centre for Integrative Neuroimaging, Department of Psychiatry, University of Oxford, Oxford, United Kingdom; ^5^Department of Neurology, Odense University Hospital, and Department of Clinical Research, University of Southern Denmark, Odense, Denmark

**Keywords:** magnetoencephalography, face perception, brain oscillations, pre-stimulus alpha activity, intra-parietal sulcus

## Abstract

Face perception is crucial in all social animals. Recent studies have shown that pre-stimulus oscillations of brain activity modulate the perceptual performance of face vs. non-face stimuli, specifically under challenging conditions. However, it is unclear if this effect also occurs during simple tasks, and if so in which brain regions. Here we used magnetoencephalography (MEG) and a 1-back task in which participants decided if the two sequentially presented stimuli were the same or not in each trial. The aim of the study was to explore the effect of pre-stimulus alpha oscillation on the perception of face (human and monkey) and non-face stimuli. Our results showed that pre-stimulus activity in the left occipital face area (OFA) modulated responses in the intra-parietal sulcus (IPS) at around 170 ms after the presentation of human face stimuli. This effect was also found after participants were shown images of motorcycles. In this case, the IPS was modulated by pre-stimulus activity in the right OFA and the right fusiform face area (FFA). We conclude that pre-stimulus modulation of post-stimulus response also occurs during simple tasks and is therefore independent of behavioral responses.

## Highlights

– Pre-stimulus alpha oscillations modulate post-stimulus response in face and object processing.
– This effect is present in simple paradigms as well as in challenging tasks.

## Introduction

Face recognition in primates, including humans, is an important brain function for all aspects of social interactions. A face conveys a large amount of information that can be processed surprisingly fast (Rossion, 2014). Numerous studies using EEG and MEG have shown a face-related component around 170 ms after stimulus onset (for a review see Yovel, 2016). In this context, the pre-stimulus oscillatory state may play an important role. Pre-stimulus activities refer to fluctuations immediately before the stimulus onset, which can affect post-stimulus processing (Milton and Pleydell-Pearce, 2016). Pre-stimulus activity may have multiple implications, including a possible role in neurofeedback techniques that are used in various settings (Braeutigam et al., 2019).

The role of pre-stimulus activity on face processing has previously been studied with several paradigms including bi-stable perception (Hesselmann et al., 2008; Peatfield et al., 2017; Rassi et al., 2019b), binocular rivalry (Hsieh et al., 2012; Rassi et al., 2022), under conditions where faces emerge from noise (Esterman and Yantis, 2010) and also in task designs that benefit from cueing of the incoming stimuli category (Puri et al., 2009). All of these studies involve a challenging task condition. For example having to choose between two completely different images, where the perception of one negates the presence of the other. They have also used behavioral data (reaction time and accuracy) to demonstrate the pre-stimulus effects (Hesselmann et al., 2008; Esterman and Yantis, 2010). Even though these studies have shed light on the importance of pre-stimulus oscillations, in a real-world environment we are confronted with a mixture of challenging and simple task conditions. Simple task conditions may not necessarily yield behavioral differences due to ceiling effects. Additionally, pre-stimulus brain activities may affect the intermediate post-stimulus neural activities, but by the time that the behavioral decision is made, the relation between pre-stimulus and post-stimulus neural activities is not visible in behavior or it may be mixed with other factors (Lou et al., 2014). This is especially true about adult participants as variability in behavioral performance is found to decrease with an increase in age (Mcintosh et al., 2008).

Previous studies found that pre-stimulus activity mainly mattered in brain regions that are crucial for one stimulus type, but not for the other (Peelen and Kastner, 2011; Ruhnau et al., 2014). In the context of face processing, essential regions are mainly in the right hemisphere and include the fusiform face area (FFA; Hesselmann et al., 2008; Rassi et al., 2019b), the occipital face area (OFA; Peatfield et al., 2017), and the superior temporal sulcus (STS; Esterman and Yantis, 2010). In addition, the lateral occipital complex (LOC) is recognized as an object-selective region, and pre-stimulus activity in LOC was found to affect facial and non-facial processing (Peatfield et al., 2017). Pre-stimulus alpha power in each of these brain regions may alter evoked responses in various parts of the brain including the frontoparietal network (FPN; Esterman and Yantis, 2010; Hanslmayr et al., 2013).

In this study, we aimed to assess the effect of pre-stimulus activity on post-stimulus face processing and compared it with non-face stimulus processing using a one-back working memory task. To this end, we have focused on alpha oscillations, as the most salient oscillation (Klimesch et al., 2007). Alpha power has been frequently suggested to be a fundamental parameter to brain states when incoming stimuli are processed (Sadaghiani and Kleinschmidt, 2016). It is noted that although evoked responses have been investigated in the context of face/non-face stimulus processing (Rossion, 2014; Besson et al., 2017), the effect of pre-stimulus activity on evoked responses has not. Here, we addressed the following questions: (1) Does a pre-stimulus effect on stimulus processing exist, even without a significant behavioral effect? (2) Is the pre-stimulus effect on stimulus processing different for face vs. object processing? and (3) Which regions of interest (ROI) show the modulatory effect of pre-stimulus alpha power on stimulus-evoked responses?

We note that it may not be possible to identify “pure” pre-stimulus brain states and some interference between pre- and post-stimulus activity might always be present. The task employed here, however, evokes only rather short-lived responses typically lasting less than 500 ms, making an overlap between the activity driven by the preceding stimulus and the subsequent pre-stimulus activity negligible.

## Method

### Participants

Twenty-two healthy individuals (21 males, 20 right-handed, 24 ± 5 years old on average) participated in the experiment. Written informed consent was obtained from all subjects. All procedures were in compliance with the Code of Ethics of the World Medical Association (Declaration of Helsinki) and approved by the Oxford Research Ethics Committee B (Ref. 07/H0605/124).

### Experimental Procedure

First, a red fixation point was displayed for 1.6 ± 0.3 s (uniform jitter). Then, a static, gray-scale image was presented. The onset of this image presentation is considered the trial onset throughout this study. All images were standardized for visual angle (8 × 6 degrees) and luminosity (42 ± 8 cd/m^2^) and were displayed for 200 ms. Subsequently, the screen was blanked for 1.2 ± 0.3 s and followed by another image of the same stimulus type. Participants were asked to press a button within the next second using their right index finger if the second image was a repetition of the first one. Otherwise, the button under the right middle finger indicated that the second image was different from the first. Button presses were delivered by an optical-fiber feedback system to the MEG system. Participants’ reaction time (RT) was measured as the difference between the time of the button press and the stimulus onset of the second image. Image pairs comprising human faces, monkey faces, and motorcycles were presented in random sequence, with 48 trials per category. In half of the trials, the first and second images were identical and in the other half, they were different. However, the stimulus category always remained the same within each trial.

### Data Acquisition

Neuromagnetic responses were recorded with the VectorView^TM^ MEG system at Oxford Centre for Human Brain Activity. The system features a helmet-shaped array of 102 detector triplets, each containing pairs of planar first-order gradiometers and a magnetometer. The outputs of each pair of gradiometers are most sensitive to the tangential current flow in the region directly below the detectors, where the local root-mean-square (RMS) signal summed over the two readings is proportional to the current strength. In contrast, magnetometers are more sensitive overall but cannot differentiate between near and distant sources. The data were sampled at 1,000 Hz (0.03–330 Hz bandwidth). Physiological artifacts were identified by additionally recording two electrooculograms and an electrocardiogram. The head position was measured once before each recording. Data in this study may be available upon request to Dr. Sven Braeutigam subject to terms and conditions of the University of Oxford and relevant Ethical Committee approval.

### Data Preprocessing

Data were preprocessed using the default parameters of the signal space separation (SSS) noise reduction method implemented in Maxfilter (MaxfilterVersion 2.2.15, ElektaNeuromag, Helsinki). Using Maxfilter, we reduced magnetic interferences and transformed all MEG data of each participant to the head position of their first blocks. Further analyses were performed using the FieldTrip toolbox for EEG/MEG-analysis, version 20171111 (Oostenveld et al., 2011). The data were segmented separately for post-stimulus and pre-stimulus brain activities. We extracted epochs from −300 ms to 1,000 ms with respect to the stimulus onset. For each trial, the pre-stimulus time interval was separately segmented from −1,300 ms to 0 ms. Subsequently, data were low-pass and then high-pass filtered with cutoffs at 150 Hz and 0.5 Hz respectively by applying a single-pass, zero-phase windowed since FIR filter and Kaiser window setting to maximal pass-band deviation of 0.001 (0.1%). The number of coefficients was 93 for the low-pass and 3,625 for the high-pass filters, respectively. Before applying the filter in preprocessing, the selected epochs of raw data were padded up to a total length of 10 s by rereading 10 s of raw data from the original file. After filtering the epochs were shortened again to their original length of 1.3 s.

We used a Fieldtrip built-in method to remove SQUID jumps. In order to reduce heartbeat (ECG) and eye movement (EOG) artifacts, we computed an independent component analysis (ICA) for each data set and visually inspected the components’ time courses and topographies. We removed the contaminated components, eliminating a maximum of our components per participant. For the localization of brain activity, we transformed the mixed channel type data into a pure magnetometer system by replacing each planar gradiometer with two magnetometers computed either as the sum or the difference of the magnetometer signal at the same location and half of the gradiometer signal multiplied with the distance between the two gradiometer loops. This resulted in a new data set comprising 510 channels. The use of the combined data is superior in the signal-to-noise ratio, but otherwise, no strong differences for instance with respect to deep and shallow sources are to be expected if we had selected just the set of 204 gradiometers or 102 magnetometers instead. This is due to SSS filtering which tends to equalize the information content across types of channels (Garcés et al., 2017).

### Defining Regions of Interest

Based on the literature, we defined our ROIs, which were used as seeds or targets. Seed ROIs included face selective regions in the right hemisphere (OFA, FFA, STS) and object selective region (LOC). Although face processing is primarily right lateralized (Frässle et al., 2016), we also considered left OFA and left FFA as seed ROIs. Target ROIs included the same set of regions as the seed ROIs as well as two regions in the FPN. In fact, multiple regions in FPN have been found to be modulated by pre-stimulus activities and this has been assigned to various top-down or bottom-up effects (Lamy and Kristjánsson, 2013; Sadaghiani and Kleinschmidt, 2016). For simplicity, in this study, we only added two regions of the FPN to our set of target ROIs which are the intra-parietal sulcus (IPS) and the frontal eye field (FEF) and are thought to integrate bottom-up and top-down effects (Zelinsky and Bisley, 2015). Additionally, in order to validate the specificity of effects, we selected the left inferior frontal gyrus (IFG) as the control region and added it to the seed ROIs. The left IFG is an important brain region in language processing and is expected to get involved when processing sentences or groups of words, but not for single words (van der Burght et al., 2019). Thus, we hypothesized that pre-stimulus alpha power in the left IFG should not have an effect on evoked responses in the target ROIs.

To solve the inverse problem, we first created the head model and the source model. To this end, for each participant, we scaled the skin surface of the Colin27 template to closely match the individual fiducial and digitization points using an affine transformation with a total of nine (three shifts, three linear scaling, and three rotations) parameters. This gives us a 4 × 4 scaling matrix per participant. Later, we used the realistic BEM head model of the Colin27 template and applied the same participant-specific transformation to it to create the volume conductor model for that individual. In the second step to build the source model, we transformed the inner skull surface of the Colin27 model using the participant-specific transformation and an additional shrinking of 12 mm. Twelve millimeter is considered a realistic approximation of the mean gray matter depth. This way the identical model mesh was geometrically individualized and all grid points were always placed inside the brain template. Individual leadfield matrices were computed for each participant based on the individual head and source models and the sensor positions of the respective measurements.

In the next step, we located our ROIs on the Colin27 brain template surface (Holmes et al., 1998). For each ROI, we assigned a center of gravity according to the published coordinates in previous studies, namely for the OFA (Pitcher et al., 2011), the FFA and the rSTS (Harris et al., 2012), the LOC (Hanslmayr et al., 2013; Peatfield et al., 2017), the IPS and the FEF (Marois et al., 2004; Kim, 2014), and the left IFG (Ardila et al., 2016). Each ROI consisted of all vertices within a diameter of less than 10 mm to its center of gravity.

In order to localize sources, we selected the linearly constrained minimum variance (LCMV) beamforming technique which projects the measured field distribution into the brain such that it preserves the activity at the region of interest while attenuating activity from all others (Van Veen et al., 1997). We have selected this method because we had a clear idea about the regions we would like to investigate. To create the spatial filter using the LCMV beamformer method, we first computed the covariance matrix of the sensor space data. The covariance matrix was computed over the pre-stimulus interval, i.e., from −1.3 s to 0 based on the data of all three stimuli types. Computing the covariance matrix based on the pre-stimulus interval in beamforming technique was also performed in previous studies (Rassi et al., 2019a, b). This covariance matrix was used to compute the spatial filter applied to pre- and post-stimulus data. Using the same covariance matrix for all conditions results in a fair reference for all comparisons because the condition differences cannot stem from differences between the covariance matrices. We used the default procedure in Fieldtrip for computing the covariance matrix in which the covariance for each individual trial was computed first and then the final covariance was estimated as the sum over all trials normalized by the total number of samples in all trials. The rank of this covariance matrix-matched the rank of the raw data. The inverse of the covariance matrix was computed by the default procedure in Fieldtrip in which the “pinv” function and the default regularization were applied. The spatial filter was also computed using the default procedure in Fieldtrip in which they computed PINV/SVD to cover rank deficient leadfield and referenced it to equation number 23 in Van Veen et al. (1997). We used the created spatial filter for each ROI to project from channels into the brain (source space). This utilizes a demixing which is superior to the channel data if the model approximates the real shape. The created spatial filter was applied to both pre- and post-stimulus sensor space data yielding three orthogonal time-courses per ROI.

### Source Analysis

**Figure 1:**
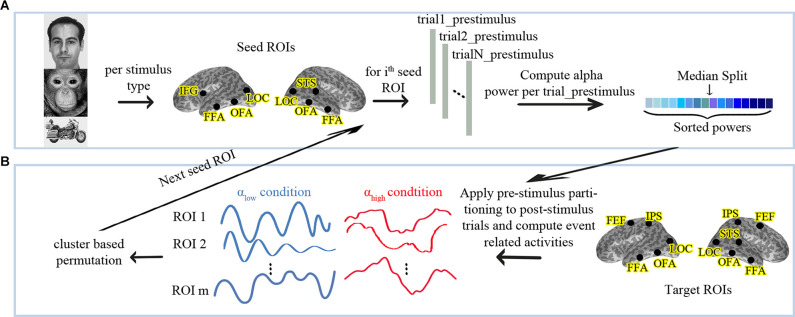
**(A)** Analysis pipeline for partitioning the data into high vs. low pre-stimulus alpha conditions and **(B)** follow-up statistics. α_low_: Low Pre-stimulus Alpha. α_high_: High Pre-stimulus Alpha. The human face image used in this figure is from the FERETopen source database (Phillips et al., 1998).

Time-frequency analyses were performed using a Morlet wavelet for the pre-stimulus interval from 1,300 ms to 0 ms using second-order detrending, zero-padding (to 4,000 ms), a wavelet width of seven cycles, in time steps of 25 ms, and frequency steps of 1 Hz. This resulted in a four-dimensional matrix (trials, ROIs, frequency bins, time bins) in which the power was computed for each trial, three time-courses of each ROI, each frequency bin, and each time bin. Alpha (8–13 HZ) power was separately estimated for each trial and ROI as the mean over the power values of the relevant frequency bins, all time bins and all the three time courses. Next, we divided the trials into two conditions based on the median of the pre-stimulus alpha power in each of the seed ROIs. This has been done separately for each participant’s data set and led to the creation of the two conditions; low and high pre-stimulus alpha power (α_low_ vs. α_high_). Figure 1A represents the partitioning pipeline in this study. The same categorization was applied to the post-stimulus periods of all first stimuli. We averaged the event-related activity following the first stimuli separately for both conditions, all target ROIs and the three stimulus categories. The second stimulus presentations are not reported here as they represent an even longer pre-to-post-stimulus time interval and additionally depend on the processing of the first stimuli.

### Statistical Analyses

We performed a nonparametric cluster-based permutation test between the α_low_ and the α_high_ conditions using a dependent-sample permutation *t*-test (Maris and Oostenveld, 2007) to evaluate whether pre-stimulus alpha in the seed nodes differentiates evoked responses in any of the target ROIs. In permutation tests, trials are repeatedly and randomly split into two groups and the statistics are re-conducted to obtain a proper null distribution. A cluster-based permutation is an appropriate approach to solve the multiple comparison problem when the spatiotemporal locus of a possible effect is not known in advance (Maris and Oostenveld, 2007). In more detail, instead of considering a separate H0 for each pair of (ROI, time point) samples, cluster-based permutation combines the neighboring samples into clusters and compares these clusters vs. the randomized distribution of clusters. The procedure is motivated by the observations that neighboring samples and space are not completely independent of each other, and the combination of effects into one cluster accounts for that. Temporal neighborhood is simply given by the sampling sequence. Spatial neighborhood needs to be defined based on a distance measure. ROIs at close distances are considered neighbors, while ROIs at larger distances remain unrelated. Because OFA and FFA are at a spatially close distance in the brain, we defined them as neighbors in each hemisphere. Further details about the permutation analyses were as follows. We applied a Monte-Carlo method for cluster-based permutation on the evoked responses of all target ROIs from 0 ms to 500 ms after the stimulus onset with 5,000 draws. Temporal and spatial adjacent samples whose *t*-values exceeded the threshold *p*-level of 0.05 (uncorrected) were combined in the same cluster. Cluster level statistics were calculated by taking the sum of the t-values within every cluster and then considering the maximum of the cluster-level statistic. The cluster-level statistic was corrected for multiple comparisons using an FWER threshold of 0.05 for the stimuli types and the two-tailed *t*-test. Figure 1B displays the described procedure.

## Results

### Behavioral Response

The mean behavioral accuracy was 90% for human face stimuli, 82% for monkey face stimuli, and 85% for motorcycles stimuli. For each seed ROI, we ran separate *t*-tests over the reaction times (RT) of the two conditions (α_low_ and α_high_) per stimulus category and found no significant difference. Table 1 shows the result of group comparisons.

**Table 1:** *p*-values from *t*-tests over the reaction times (RT) of the two conditions (low vs. high alpha power) per stimulus category.

	Human faces	Monkey faces	Motorcycles
Right FFA	0.76	0.08	0.38
Left FFA	0.67	0.34	0.12
Right OFA	0.56	0.06	0.33
Left OFA	0.75	0.32	0.14
Right STS	0.96	0.10	0.09
Right LOC	0.68	0.13	0.18
Left LOC	0.58	0.11	0.13
Left IFG	0.64	0.87	0.31

### Cluster-Based Permutation Over the Evoked Responses

**Figure 2:**
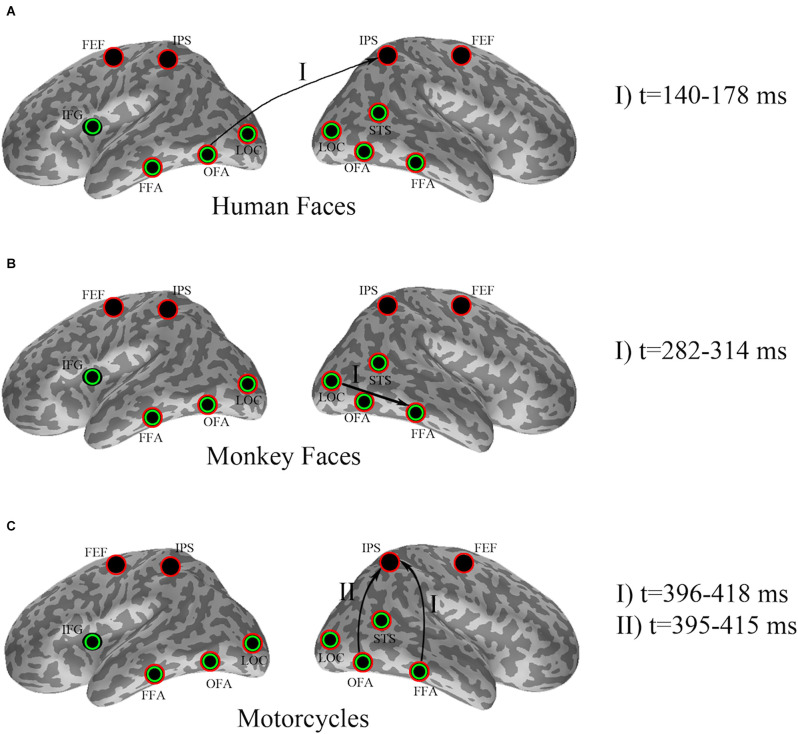
Effect of pre-stimulus alpha power on evoked responses for **(A)** human faces, **(B)** monkey faces, and **(C)** motorcycles. Seed ROIs were marked with green circles, whereas red circles specified the target ROIs. Arrows indicate significant effects, where pre-stimulus alpha-power in a seed region (beginning node in each directional connection) modulates the evoked response in a target region (ending node of that connection). The time intervals at which the significant dissociations were recognized by cluster-based permutation have been added as the legend.

Descriptive statistics for the effects found by cluster-based permutation are illustrated in Figure 2.

**Figure 3:**
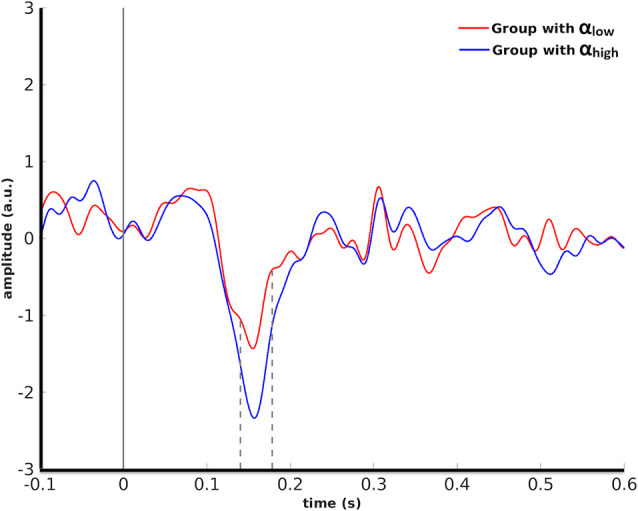
Grand average over the participants’ evoked responses to human faces in the right IPS for the α_low_ vs. the α_high_ conditions. Dashed lines indicate the time interval at which the difference between the two conditions was found to be significant by cluster-based permutation.

In detail, cluster-based permutation analyses revealed that for human faces, the left OFA was the only seed ROI whose pre-stimulus alpha power yielded a significant dissociation in the target ROIs between the evoked responses of the two conditions (*p* = 0.034). This effect was exposed in the right IPS from 140 ms to 178 ms after stimulus onset such that the condition with high pre-stimulus alpha power had a significantly larger amplitude than the α_low_ condition (Figure 3).

**Figure 4:**
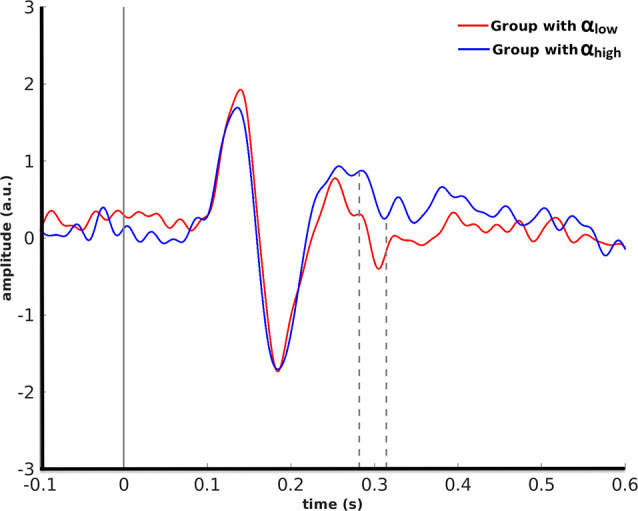
Grand average over the participants’ evoked responses to monkey faces in the right FFA for the α_low_ vs. the α_high_ conditions are partitioned based on pre-stimulus alpha power in the right LOC.

A cluster-based permutation for monkey face stimuli showed that pre-stimulus alpha power in the right LOC yielded significant effects (*p* = 0.01) in target ROIs. This effect was emerged in the right FFA from 282 ms to 314 ms after stimulus onset by significantly lower amplitude for the α_low_ condition in comparison to the α_high_ condition (Figure 4).

**Figure 5:**
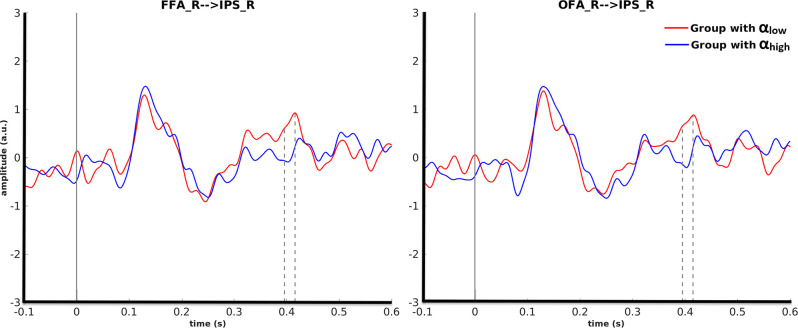
Grand average over the participants’ evoked responses to motorcycles stimuli for the α_low_ and α_high_ conditions in the right IPS. The left columns represent the time courses when FFA was considered as the seed ROI and the right column is with regard to OFA as the seed ROI.

Cluster-based permutation revealed that for motorcycle stimuli partitioning the data based on pre-stimulus activity in the right OFA could discriminate between evoked responses in the right IPS from 395 ms to 415 ms after (*p* = 0.045). A similar effect was found in the right IPS from 396 ms to 418 ms after stimulus onset when cluster-based permutation was performed based on pre-stimulus alpha power in the right FFA (*p* = 0.035). For both of these effects, the amplitude in the right IPS was smaller for α_high_ than for α_low_. Further investigation showed that 87% of trial assignments based on pre-stimulus activity (α_low_ vs. α_high_) in the right OFA and the right FFA were identical (Figure 5).

Considering the left IFG as the control region, there was no significant effect on target ROIs for any of the stimuli types.

## Discussion

In this study, we investigated the role of pre-stimulus alpha power on event-related activity after showing the participants three types of stimuli: the human face, monkey face, and motorcycle. Previous studies showed that the perception of monkey faces recruits similar neural mechanisms as the perception of human faces (Rousselet et al., 2004). In the current study, we examined how comparable they are in terms of the effect they received from pre-stimulus oscillations. Pictures of motorcycles were also shown to the participants as objects with similar spatial frequency distribution to that of human faces (Kylliäinen et al., 2006). We showed that a significant difference in stimulus-evoked activity modulated by pre-stimulus alpha power, but without a corresponding behavioral response. Interestingly, the effect of pre-stimulus alpha depends on the stimulus category, suggesting that pre-alpha interacts in a specific way with the stimulus-driven activity in face and object processing networks.

Alpha oscillations have been reported to be associated with an inhibitory gating mechanisms (Klimesch et al., 2007; Haegens et al., 2011; Hanslmayr et al., 2013). in situations where there is a competition between two stimuli leading to two different perceptions, an increase of alpha power in brain regions, which are not critical for one stimulus type (but usually is for the other stimulus type) has been found to facilitate the processing of the former stimulus. For instance, in a bi-stable task design in which participants had to choose between perceiving the presented stimulus as a face or vase, the increase of alpha power in the LOC before stimulus onset was correlated with subject perception as the face (Peatfield et al., 2017). Our results indicated that pre-stimulus alpha oscillation in the left OFA resulted in a significant difference between post-stimulus activity in response to human face around the M170 component which is a prominent component in face perception (Rossion, 2014) in the right IPS. Considering that face processing is right-lateralized (Frässle et al., 2016), some studies (Knakker et al., 2015) have shown that attending to a face stimulus would increase alpha power over left parieto-occipital regions. Although no source localization was performed in their study to precisely locate the regions involved, this could be taken as evidence for regarding the left OFA as a non-specific region for face processing. Higher pre-stimulus power in the left OFA thus can be attributed to more inhibition of a face non-specific region. On the other hand, the α_high_ condition was found to be associated with a larger activity in the right IPS around the M170 component in contrast to α_low_ condition. This supports the model that inhibiting activity in a non-specific region (the left OFA) facilitates activity in the relevant regions (the right IPS).

Using event-related activities, we showed that for monkey face stimuli the pre-stimulus alpha variations in the right LOC lead to a significant separation of post-stimulus processing from 282 ms to 314 ms after stimulus onset resembling the face selective M250 component (Nasr and Esteky, 2009; Olivares et al., 2015). This effect emerged in the face selective region of the right FFA where the α_high_ condition had higher amplitude compared to α_low_. LOC has been reported as a face non-specific region in multiple studies (Hesselmann et al., 2008; Peatfield et al., 2017). Hence, again more pre-stimulus alpha power in a non-specific region (the right LOC) apparently translates to improved processing in a face specific region (the right FFA).

Pre-stimulus impact on post-stimulus processing of motorcycles was observed around M400 in the IPS. Seed ROIs for these effects were the right OFA and the right FFA, which are considered as motorcycle unselective ROIs. Thus, the increased power of pre-stimulus alpha activity in these seed ROIs should facilitate post-stimulus processing. However, this expectation does not lead to a higher amplitude for α_high_ in comparison to α_low_ in IPS. This suggests that while the inhibitory role of pre-stimulus alpha is evident in earlier components, later components may be independent of the pre-stimulus alpha level or may work *via* a different mechanism. Interestingly, a recent study (Iemi et al., 2019) reported that pre-stimulus alpha activity could affect late components in a reverse manner in contrast to the early event-related components due to the baseline shift.

In our analysis, we also partitioned the data based on pre-stimulus alpha power in the left IFG. Left IFG is activated in more complex language processing and thus irrelevant with regard to our experiment. Thus, we expected that the level of pre-stimulus alpha power in left IFG would not yield a significant dissociation of evoked responses in the target ROIs and our results affirmed this hypothesis. This is an evidence in favor of the specificity of our effects and indicates that the inhibitory role of pre-stimulus alpha power is mainly expressed in brain regions which are accounted as important regions in processing of the other stimulus type.

The cognitive effect associated with pre-stimulus alpha oscillations is not entirely understood. Using specialized task designs, previous studies assigned pre-stimulus alpha oscillations in stimulus/task selective regions to various bottom-up or top-down cognitive concepts including preparatory selective attention (Battistoni et al., 2017), prediction (Summerfield and Egner, 2009), priming (Theeuwes, 2013), and consciousness (Peatfield et al., 2017). While our task design does not allow us to directly attribute the pre-stimulus activity to a specific cognitive function, we offer some speculations here based on previous reports.

*Preparatory selective attention* in the pre-stimulus interval is a form of spatial, or feature-based, selective attention and acts by selective pre-activation of the relevant features (including stimulus type or locations) before stimulus presentation (Battistoni et al., 2017). However, this is known to be true only when a cue is used before stimulus onset to inform the participant about the feature(s) or location(s) of the incoming stimulus. Considering the fact that we did not use any cue in the pre-stimulus interval, the effects we have seen in our results could not be ascribed to preparatory attention.

*Prediction* is a term, which has been used with two different meanings in the literature. Some studies have stated that ascribing pre-stimulus alpha activation in stimulus/task sensitive regions to prediction is only possible when the probability of occurrence of stimuli is different over the trials (Summerfield and Egner, 2009). In our task design, the probability of the presence of human faces, monkey faces, and motorcycles are equal, thus the pre-stimulus oscillations in the seed ROIs in our study may not be explained by this kind of *prediction*. Some studies stated that variability in pre-stimulus fluctuations that bias post-stimulus activities and behavioral responses may be a type of prediction (Hsieh et al., 2012), even though the probability of stimuli presentation was equal in their study. In the latter form, we could attribute the effect that we saw in our study as *prediction*.

Variability in the characteristics of pre-stimulus oscillation has been suggested to play a crucial role in *conscious visual processing* (Peatfield et al., 2017), usually defined in terms of the ability to recall and actively report an object or image previously seen (Dehaene and Changeux, 2011). Moreover, this putative role of pre-stimulus activity in conscious processing is broadly in line with evidence obtained from attentional blink studies suggesting that failure to consciously perceive a target stimulus (so-called T2) is related to an increase in alpha and low beta oscillatory activity at 80–140 ms after onset of the preceding (so-called T1) stimulus (Slagter et al., 2017). This is neither an attentional blink study, nor the subjects “blinked” to the stimuli, however, one could speculate that the effects observed here related to some extent to the mechanisms associated with conscious visual processing, where pre-stimulus alpha modulates attention while attending a sequence of stimuli. This view is supported by the late effect seen for motorcycles suggesting that conscious processing commences within 300 ms after stimulus onset (Dehaene and Changeux, 2011; Navajas et al., 2013; Rossion, 2014), and the well-documented role the IPS plays in consciousness (Dehaene et al., 2006). Moreover, although the response to the human face peaks at around 170 ms and for the monkey face from 282 ms to 314 ms after stimulus onset, several previous studies suggested that the conscious process for face processing may occur in less than 300 ms (Navajas et al., 2013; Rossion, 2014).

*Priming* is defined as a non-conscious influence on cognitive processing (Dudai, 2004) which manifests itself 300 ms after stimulus presentation (Dehaene and Changeux, 2011), and inter-trial priming could happen in a variety of situations (Theeuwes, 2013). Therefore, the effect related to motorcycles in our results, which emerged after 300 ms is unlikely to be a priming effect. For face stimuli, the response occurred in less than 300 ms and therefore the pre-stimulus effect could be attributed to priming.

Our study showed that the key region for the effect of pre-stimulus activity on post-stimulus response is the IPS, which is thought to be responsible for concatenating top-down and bottom-up information. The FEF has a similar function to that of IPS but we saw no effect in the FEF. This is consistent with a previous study that the presence of an effect in the FEF is associated with a behavioral change but the response in the IPS alone is not necessarily related to a behavioral change (Mirpour and Bisley, 2012). Thus, the absence of any effect from pre-stimulus oscillation on the post-stimulus activity in the FEF is compatible with the lack of behavioral differences in our study.

Contrary to other stimuli types, the effect found for monkey faces was in the FFA, which is thought to encode second order structural configurational information, particularly in the time frame that it occurred in our study (Nasr and Esteky, 2009). Interestingly, we observed that the transfer of information from pre-stimulus to post-stimulus was faster for human face processing than that of monkey face. In addition, unlike for human faces, we found an inhibitory role of the pre-stimulus alpha activity of the LOC on monkey face response. We propose that this difference in our study is due to the expertise of human subjects in processing human faces compared to monkey faces (Pascalis and Bachevalier, 1998). Contrary to our findings, previous studies (Hesselmann et al., 2008; Peatfield et al., 2017) reported this effect of the LOC activity on the human face, however, their task was more challenging than ours (Pascalis and Bachevalier, 1998).

## Conclusion

This study provided support for the notion that pre-stimulus activity has an important modulatory role in post-stimulus processing and that this effect is stimulus selective. In addition, the pre-stimulus effect does occur in a simple task mainly in the IPS, and is not associated with an apparent behavioral response. The absence of individual MRI scans was a limitation of this study which restricted the source analysis to the use of an individually scaled standard model and did not allow to take advantage of the individual cortical folding during source localization. Additionally, the absence of significant behavioral effects as a function of the differences in pre-stimulus alpha power does not allow us to show the behavioral consequences of the observed differences in certain brain areas. The observed differences, however, match previous findings for more challenging tasks thereby letting us conclude that different pre-stimulus alpha power levels very likely modulate later brain activity regardless of the behavioral consequences.

## Data Availability Statement

The raw data supporting the conclusions of this article will be made available by the authors, without undue reservation.

## Ethics Statement

The studies involving human participants were reviewed and approved by Oxford Research Ethics Committee. The patients/participants provided their written informed consent to participate in this study.

## Author Contributions

SB contributed to conception and design of the study, organized data collection. ND and BM performed source analysis and the statistical analysis. All authors contributed to the interpretation of the results. ND wrote the first draft of the manuscript under supervision of BM, RK, SB, and MZ. All authors intellectually contributed to the article and approved the submitted version.

## Conflict of Interest

The authors declare that the research was conducted in the absence of any commercial or financial relationships that could be construed as a potential conflict of interest.

## Publisher’s Note

All claims expressed in this article are solely those of the authors and do not necessarily represent those of their affiliated organizations, or those of the publisher, the editors and the reviewers. Any product that may be evaluated in this article, or claim that may be made by its manufacturer, is not guaranteed or endorsed by the publisher.
